# Contrasting associations between parental coordination and breeding success in albatrosses across differing environmental contexts

**DOI:** 10.1111/1365-2656.70350

**Published:** 2026-09-15

**Authors:** Natasha Gillies, Richard A. Phillips, Henri Weimerskirch, Jonathan R. Potts, Denis Réale, Alastair J. Wilson, Frédéric Angelier, Christophe Barbraud, Ashley Bennison, Karine Delord, Prescillia Lemesle, Samuel Peroteau, Andrew G. Wood, José C. Xavier, Samantha C. Patrick

**Affiliations:** ^1^ School of Environmental Sciences University of Liverpool Liverpool UK; ^2^ British Antarctic Survey, Natural Environment Research Council, High Cross Cambridge UK; ^3^ Centre d'Études Biologiques de Chizé, UMR 7372 du CNRS‐La Rochelle Université Villiers‐en‐Bois France; ^4^ School of Mathematical and Physical Sciences University of Sheffield Sheffield UK; ^5^ Département des sciences biologiques Université du Québec à Montréal Montréal Quebec Canada; ^6^ Centre for Ecology and Conservation University of Exeter, Penryn Campus Cornwall UK; ^7^ Associate Laboratory TERRA, Department of Life Sciences Centre for Functional Ecology (CFE), University of Coimbra Coimbra Portugal

**Keywords:** behavioural flexibility, biparental care, environmental variability, foraging behaviour, parental investment, reproduction, seabirds

## Abstract

1. Understanding how animals acquire resources and allocate them to reproduction is fundamental for identifying the factors shaping fitness. In species with biparental care, both the amount each parent invests and how well their efforts are coordinated can influence reproductive success. Yet the benefits of coordination are unlikely to be universal and instead may depend strongly on species' ecology and environmental context. Despite this, many studies still focus on one parent in isolation, overlooking the potential importance of joint investment strategies.
2. Here, we examined how parental effort and coordination, measured here as the relative investment of both parents, influenced breeding success in black‐browed *Thalassarche melanophris* and wandering albatrosses *Diomedea exulans* across colonies characterised by differing levels of environmental variability.
3. We found that the influence of parental coordination on breeding success differed between species, breeding stages, and sites. Longer foraging trips improved annual breeding success during incubation for black‐browed albatrosses, whereas the effect of longer trips during brooding varied between sites subject to differing environmental variability.
4. Similarity in the predictability and duration of partners' foraging trips was associated with higher breeding success at sites with higher environmental stability in both species but had little effect where conditions were more variable. This suggests that patterns of reproductive investment may differ according to environmental context, potentially reflecting a shift toward individual flexibility in more unpredictable conditions.
5. Our findings underscore the importance of considering both parents' contributions when linking reproductive strategies to breeding outcomes and highlight how observed differences in breeding success across colonies may reflect shifts in the trade‐offs between self‐maintenance and offspring investment within varying environmental contexts.

## Graphical abstract

Our study demonstrates that the benefits of parental coordination in albatrosses are context‐dependent rather than universal. Coordinated parental care enhanced reproductive success in stable environments, but had little effect in variable environments, where individual flexibility may be more valuable. These findings highlight how ecological unpredictability shapes the trade‐offs between joint investment and fitness.

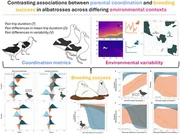

## 1 INTRODUCTION

Identifying the factors that drive variation in fitness of individuals among populations is a central problem in evolutionary biology. Such variation arises from both intrinsic individual differences and extrinsic ecological and environmental factors, the latter of which can shape the former. Together, these influence how individuals acquire and allocate resources between reproduction and survival, shaping fitness and driving evolutionary trajectories. Although much research has sought to quantify individual variation in these processes, studies often overlook that in sexually reproducing animals, especially those with biparental care, offspring survival—which over a lifetime is the primary component of fitness—depends on two individuals (Black & Hulme, 1996; Griffith, 2019; Kölliker, 2012). As a result, fitness depends not just on the behaviour of the individual but also on that of its partner and their interactions (Johnstone et al., 2014; Mariette & Griffith, 2015; Wolf et al., 1999; Zheng et al., 2025).

In animals, behaviour is one of the main mechanisms by which intrinsic and extrinsic variation is translated into fitness (Bateson, 2004; Duckworth, 2009; Plotkin, 1988), providing a pathway for integrating multiple factors that determine survival and reproduction. One key example is optimal foraging theory, which predicts that individuals should behave in such a way that optimises resources gained for a given time investment, and through this maximise their fitness (Charnov, 1976). Indeed, many studies have sought to investigate the links between individual foraging behaviour and reproductive success (Frey‐Roos et al., 1995; Jeanniard‐du‐Dot et al., 2017; Kowalczyk et al., 2015; Lemon, 1991; Trevail et al., 2019). However, few of these have examined how the behaviours of the partners combine to influence these relationships (Kavelaars et al., 2021; Mariette & Griffith, 2015; Mutzel et al., 2013).

In species where the lifetime reproductive output of partners is likely to be closely linked—for example because the costs of divorce are very high (Choudhury, 1995; Culina et al., 2015), because individuals typically engage in lifetime monogamy (Roughgarden, 2012), or because there is a benefit to mate familiarity (Naves et al., 2007; Sanchez‐Macouzet et al., 2014)—the situation is further complicated because parents may be selected to distribute the costs of care across the partnership (Griffith, 2019). This contrasts with systems in which mate change is more frequent and future reproduction is less dependent on pair bond duration; under such circumstances, there may be less incentive to compensate for lower effort by the partner (Johnstone & Hinde, 2006; McNamara et al., 2000). In this context, variation in individual reproductive performance, investment or resource acquisition may be masked if pair members compensate for or coordinate with one another (Ihle et al., 2019; Roth et al., 2021; Spoon et al., 2006). Fitness, therefore, manifests as an emergent property of the combined efforts and interactions of the pair, as the level of cooperation or coordination may itself be a source of selection (Spoon et al., 2006).

Across species and populations, distinct strategies may have evolved due to their context‐dependent fitness consequences across ecological contexts. For instance, the relative fitness payoffs of resident versus migratory individuals often depend on environmental conditions (Buchan et al., 2020), while varying environmental predictability can favour either flexible or consistent behavioural phenotypes (Mathot et al., 2012). Similarly, the behavioural strategies pairs adopt to coordinate or divide investment in offspring may be expected to vary in their fitness consequences depending on the ecological conditions a population experiences. Exploring how such pair‐constrained behaviours differentially influence breeding success across species and populations can therefore reveal the underlying mechanisms driving reproductive success. Such insights can also illuminate the extent to which individual strategies and behaviours are shaped by broader ecological forces. To address this, we examined the effects of both relative investment and coordination within pairs on reproductive success for two albatross species breeding at colonies experiencing contrasting environments. Albatrosses are long‐lived, monogamous birds that depend on dynamic ocean systems while raising offspring cooperatively with their partner (Tickell, 2000). They therefore provide an ideal study system for examining the constraints acting upon species with exceptionally long lifespans, for which breeding success is closely tied to the availability of spatially and temporally unpredictable resources (Gaston, 2004). As central‐place foragers, during the incubation and brooding periods—when one parent is constrained to remain at the nest—the main parental behaviours involve trade‐offs between time spent at sea feeding for themselves and to gather food for the offspring, versus incubating the egg or brooding the chick until it can be left unattended (the latter allows both parents to forage simultaneously at sea). While time at the nest is constrained by the need to avoid offspring mortality, variation in foraging trip duration provides a more flexible behavioural window into individual investment decisions. Consequently, some parental behaviours and investment can be meaningfully inferred from trip durations. When at the nest incubating the egg or brooding the young chick, parents fast continuously, during which they pay high energy costs that must be recouped during their subsequent foraging trip (Croxall & Ricketts, 1983; Nord & Williams, 2015; Tickell & Pinder, 1975).

To explore how these trade‐offs manifest in natural populations with differing life histories and foraging strategies, we compared the behaviour of wandering albatrosses (*Diomedea exulans*) and black‐browed albatrosses (*Thalassarche melanophris*) breeding on islands in the sub‐Antarctic Atlantic Ocean (South Georgia) and Indian Ocean (Crozet and Kerguelen islands; Figure 1). Wandering albatrosses usually breed every 2 years if successful but exhibit exceptionally high breeding success, whereas black‐browed albatrosses are usually annual breeders that show greater variation in breeding success among individuals and between oceans (Nevoux et al., 2010; Tickell, 2000). Both species lay a single egg per breeding attempt, and parents share incubation and chick‐rearing duties. In wandering albatrosses, incubation lasts for 75–80 days, the brooding period lasts c. 1 month, and chicks fledge after 7–10 months, whereas in black‐browed albatrosses, incubation lasts for 68–71 days, the brooding period lasts c. 3 weeks, and chicks fledge after 4 months (Tickell, 2000). At South Georgia, populations of both species have been declining steeply over the last few decades, with wandering albatrosses declining by 1.8%–3% per year and black‐browed albatrosses by 1.9% per year (Mackley et al., 2025; Poncet et al., 2017; Rackete et al., 2021). Beyond the well‐documented impacts of longline fisheries on albatross survival (Warwick‐Evans et al., 2026), population declines are further exacerbated by high environmental variability, including changes in sea‐surface temperature, sea ice cover and increasing wind strength (Pardo et al., 2017). Oceanographic conditions around South Georgia are highly influenced by the El Niño‐Southern Oscillation (ENSO) and Southern Annular Mode (SAM) (Murphy et al., 2007; Trathan & Murphy, 2002), leading to considerable inter‐annual variation in environmental conditions. Populations in the Indian Ocean, however, are more stable and experience a less variable environment (Rolland et al., 2009; Weimerskirch et al., 2018) driven primarily by SAM, with little influence of ENSO (Pohl et al., 2021). These environmental differences are believed to drive differences in life history parameters (Nevoux et al., 2010) and senescence rates (Mohring et al., 2026) between the populations, but their effects on behaviour are not known.

**FIGURE 1:**
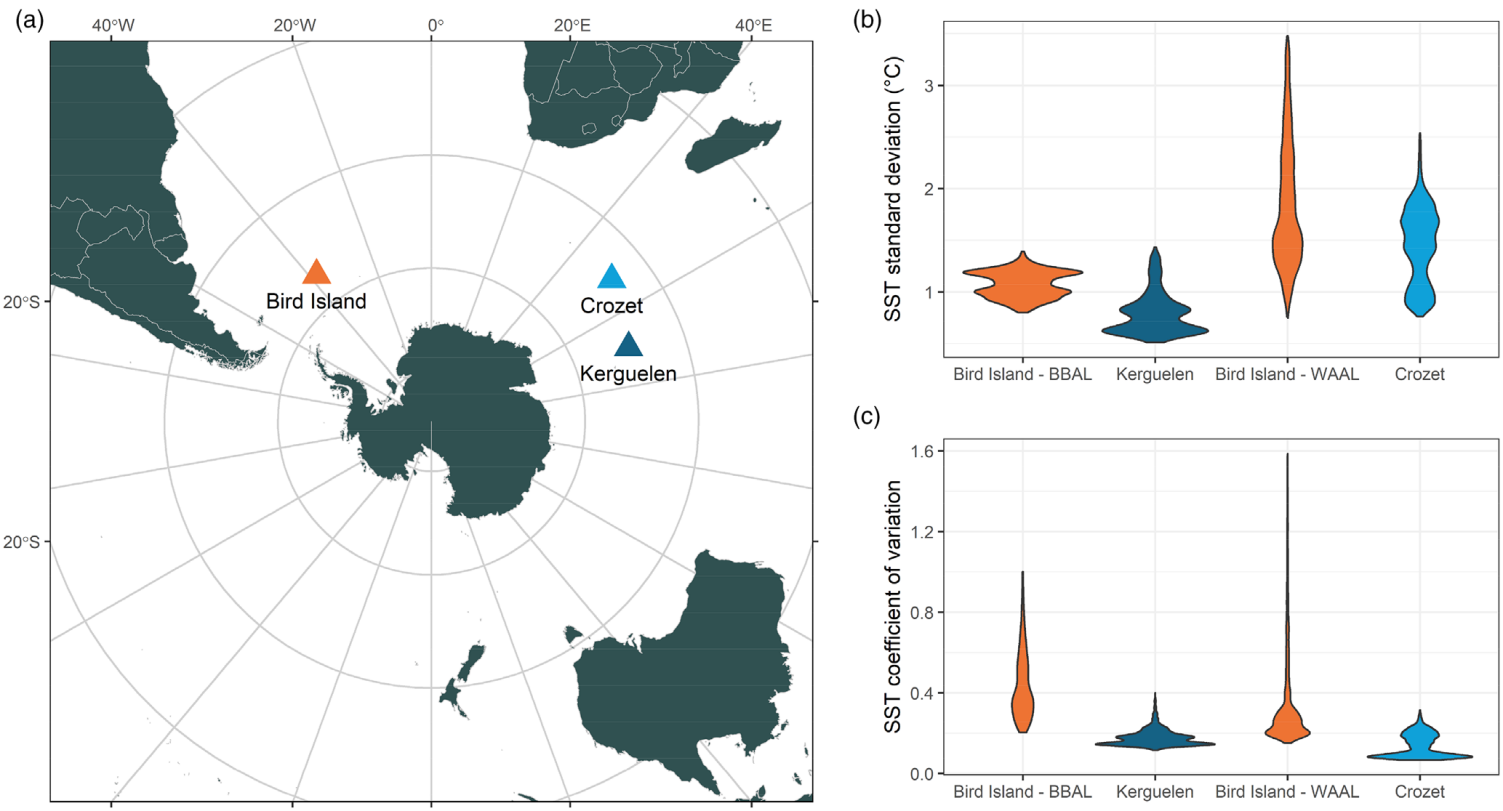
(a) Locations of the three study colonies, Bird Island, Crozet and Kerguelen, in the sub‐Antarctic. Map is displayed in a Southern Hemisphere stereographic projection centred on the South Pole. (b) Temporal standard deviation of sea‐surface temperature (SST, °C) and (c) coefficient of variation of SST across grid cells within the core foraging ranges of each population. BBAL, black‐browed albatrosses; WAAL, wandering albatrosses.

Using a 20‐year dataset, we examined wandering and black‐browed albatrosses at both islands to assess how relative parental investment and coordination influenced reproductive success. We investigated how these relationships varied between species with contrasting reproductive investment and across colonies experiencing different levels of environmental variation. We analysed three metrics (explained in detail below): (1) pair‐level median trip duration, representing overall investment in foraging; (2) within‐pair difference in trip duration, indicating the overall division of investment in foraging trips; and (3) within‐pair difference in trip variability, reflecting relative predictability of each partner and therefore coordination of foraging. We predicted that breeding success would be more positively predicted by balanced investment and coordination (i.e. parameters 2 and 3) in species and populations with more variable reproductive success and in more variable environments (Grissot et al., 2019; Saraux et al., 2011; Vincze et al., 2017). Through this, we aimed to determine the importance for breeding outcomes of the relative investment of each pair member and how well they coordinate this, and the implications for demography and, ultimately, evolutionary trajectories.

## 2 METHODS

### 2.1 Study site and field methods

Pairs of black‐browed albatrosses were tracked from Bird Island, South Georgia (54°00′ S, 38°03′ W; 76 pairs) and Cañon des Sourcils Noirs, Kerguelen Islands (49°41′ S, 70°14′ E; 173 pairs), and of wandering albatrosses were tracked from Bird Island (226 pairs) and Possession Island, Crozet Islands (46°24′ S, 51°46′ E; 163 pairs). Data were collected during the breeding seasons between austral summers 2002/03 and 2022/23 (see Table S1 for sample sizes).

All tracked birds were part of long‐term studies and were fitted with a metal ring and a plastic ring with a unique alphanumeric code for individual identification. This allowed both partners in each pair to be identified. While sex was determined for many individuals using behavioural, morphological or molecular cues, this information was not available for all birds. As a result, sex was excluded from the analyses (Table S1).

The breeding success of each pair in the study colonies was recorded as part of long‐term monitoring and was defined as a binary variable, where 1 indicates that the chick survived to fledging and 0 indicates failure at the egg or chick stage.

To validate previously reported differences in environmental variability between colonies, we quantified long‐term variability in sea‐surface temperature (SST) within the core foraging ranges of the studied populations. SST is widely used in seabird ecology as an integrative metric of oceanographic variability and is closely linked to the distribution and availability of marine prey (Carroll et al., 2015; Devney et al., 2009; Guinet et al., 1998; Pardo et al., 2017). Full methodological details are provided in the Supporting Informations and are briefly summarised here. For each colony, we extracted SST data from a spatial range reflecting the typical foraging extent of the population, derived from GPS tracking data. We then summarise temporal variability in SST within spatial grid cells and used this to compare both the magnitude and spatial heterogeneity of environmental variability across colonies. Consistent with previous studies, SST variability was higher in the foraging ranges surrounding Bird Island than around Kerguelen or Crozet (Figure 1a,b; Figure S1; Table S2).

#### 2.1.1 Tracking methods

Birds were equipped with global positioning system (GPS) and combined geolocator‐immersion (global location sensor; GLS) loggers.

For black‐browed and wandering albatrosses breeding on Bird Island, three types of GPS logger were used: i‐gotU GT‐120 (MobileAction Technology, Taiwan; 25 g, 0.8% and 0.4% body mass respectively), MiniGPSlog (earth & OceanTechnology, Germany; 25 g) and MicroGPSlog (earth & OceanTechnology, Germany; 10 g, 0.4% and 0.2% body mass). For black‐browed albatrosses breeding in Cañon des Sourcils Noirs, only i‐gotU GT‐120 was used. For wandering albatrosses breeding on Possession Island, three logger types were used: i‐gotU GT‐120/600 (weighing up to 32 g, max 0.5% body mass), X‐GPS, and Centurion (Sextant Technology, NZ; 60–75 g, max 1.2% body mass).

GPS were attached to the mantle feathers using marine Tesa® tape and were removed after the birds had completed at least one foraging trip. Previous studies found no evidence for an effect of loggers this size on survival probability or breeding success in black‐browed albatrosses (Phillips et al., 2003), or wandering albatrosses (Barbraud & Weimerskirch, 2012).

For black‐browed and wandering albatrosses breeding on Bird Island, MK19 GLS loggers (British Antarctic Survey, Cambridge, UK; 2.5 g, 0.08% and 0.04% body mass, respectively) were used. For black‐browed albatrosses breeding in Cañon des Sourcils Noirs and Possession Island, both British Antarctic Survey (Mk4, Mk5, Mk9, Mk15, Mk19) and Biotrack/Lotek (MK3006) GLS loggers were used (2.5–5 g; 0.07%–0.14% and 0.4%–0.07% body mass). GLS loggers were attached by cable tie to a plastic leg ring mounted on the tarsus and remained attached for 1–3 years.

### 2.2 Identification of foraging trips

GPS loggers were programmed to record fixes at intervals of 1–60 min. All data were resampled or interpolated to fixes at 10‐min intervals to promote comparability across the datasets. GPS data were filtered using an iterative forwards/backwards speed filter provided by the trip package in R (Sumner, 2011; Sumner et al., 2009). This removed GPS points that required unrealistic flight speeds of >80 km/h, which were ~1% of fixes across the datasets (Bird Island: black‐browed albatross—1.03%; wandering albatross—0.67%; Kerguelen black‐browed albatross—1.24%; Crozet wandering albatross—0.72%). Tracking data were split into trips based on departure and return to the colony.

If GPS data were unavailable, immersion data from the GLS loggers were used to determine foraging trip durations, an approach that has been validated in other studies (Christensen‐Dalsgaard et al., 2018; Freeman et al., 2013). The loggers tested for salt‐water immersion every 3 or 6 s and recorded the total number of immersion (wet) events every 10 or 5 min, respectively, or all transitions between wet and dry states that lasted ≥6 s. To ensure comparability between the two logger types, the data from the latter type were aggregated into 10‐min intervals. In line with previous work (Desprez et al., 2018; McCully et al., 2022; Patrick et al., 2021), we first identified periods of continuous incubation/brooding as prolonged dry periods >8 h, thus indicating the bird was on land, as only a tiny proportion of flights at sea are longer (Edwards et al., 2007). The start and end of incubation/brooding bouts were considered to be the end of the last wet bout before a dry period exceeding 8 h, and the start of the subsequent wet event, respectively. Most landings following departure are within 10 km of the colony, and so we are likely to estimate the start of trips accurately; landings before return are further away, within 100 km, but are still likely a good approximation of foraging trip duration (Kowalska O'Neil et al., 2023). While at sea, birds alternated time on the water and flights.

Our analyses considered foraging trips during the incubation and brooding periods, as it is not possible to distinguish individual trips from immersion data during the post‐brood chick‐rearing period because birds may only return to the colony for brief periods. Where possible, incubation and brooding periods were determined from observed laying and hatching dates for individuals, assuming that brooding ended a maximum of 30 days and 40 days after hatching for black‐browed and wandering albatrosses, respectively (Tickell, 1984; Weimerskirch et al., 2000). Where neither laying nor hatching date was known (15.6% pairs), we used the median hatch date for each colony and species. During incubation and brooding, while one parent is at sea, the other remains at the nest, during which time it fasts continuously. Parents of both species overlap at the nest for up to 2 h (Gillies, Guilford, & Catry, 2021; McCully et al., 2022), which is unlikely to have a substantial impact on our results given that foraging trips usually last for several days. As such, we could determine the foraging trip durations of untracked partners of tracked birds.

### 2.3 Statistical analyses

#### 2.3.1 Behavioural parameters

From the data on estimated foraging trip durations, we calculated pair‐level foraging metrics, expressed in days, that reflect both investment in parental care and coordination between partners (Figure S2). These were as follows: the median duration of foraging trips within the pair (pair‐level median trip duration), the difference in median duration of foraging trips within the pair (within‐pair difference in trip duration) and the within‐pair difference in foraging trip variability (within‐pair difference in trip variability). Trip variability was quantified as the standard deviation of all foraging trips for each parent, and the within‐pair difference was defined as the absolute difference between standard deviations. These metrics were calculated only for pairs for which data were available for each partner for at least two foraging trips (i.e. minimum four trips total).

Long foraging trips are associated with better recovery of body condition for the foraging bird in both black‐browed and wandering albatrosses (Phillips et al., 2009) but will also increase the risk of abandonment by the sitting bird if it has insufficient reserves to last until its partner returns (Cornioley et al., 2016; Pinaud & Weimerskirch, 2002; Weimerskirch et al., 2007). To measure the effect of foraging trip duration on breeding success, we used the pair‐level median trip duration, as well as the within‐pair difference in trip duration. We predicted that very long trips and large differences in trip duration between the partners would have a negative impact on breeding success, as this could force the partner to sit for longer than is optimal.

Previous studies on albatrosses showed that variability in foraging trips is associated with increased breeding failure (Patrick & Weimerskirch, 2014, 2017). We examined whether breeding failure was related to differences between partners in the variability of trip durations (within‐pair difference in trip variability). A smaller value indicates that both partners display similar levels of variability in trip durations, and a larger value that one partner is much more variable than the other. Given that individual variability reduces breeding success, we speculated that large differences in variability between the parents might also increase the risk of breeding failure.

#### 2.3.2 Modelling procedures

All statistical analyses were carried out in R version 4.3.0 (R Core Team, 2024). To examine the effect of the behavioural metrics on breeding success, we fitted binomial regression models in a Bayesian framework using the R package brms (Bürkner, 2017).

For each species (black‐browed and wandering albatross) and breeding stage (incubation and brooding), we modelled breeding success (B) as a Bernoulli‐distributed response variable:

$$B_{i}∼\text{Bernoulli}\left(p_{i}\;\right)$$

where $p_{i}$ represents the probability of breeding success. The logit of $p_{i}$ was modelled as:

$$\text{logit}\left(p_{i}\;\right)=\beta_{0}+\beta_{1}\;T_{i}+\beta_{2}\;D_{i}+\beta_{3}\;V_{i}+\beta_{4}\;C_{i}+C_{i}\left(\beta_{5}\;T_{i}+\beta_{6}\;D_{i}+\beta_{7}\;V_{i}\;\right)+U_{i}^{\text{season}}$$

where $T_{i}$ is pair‐level median trip duration; $D_{i}$ is within‐pair difference in trip duration; $V_{i}$ is within‐pair difference in trip variability; and $C_{i}$ is colony (Bird Island, Kerguelen, or Possession Island). We assessed multicollinearity by calculating variance inflation factors (VIF) for all predictors, using the R package performance (Lüdecke et al., 2019). VIF values indicated moderate multicollinearity for ‘colony’ in the brooding model for wandering albatrosses. As this likely reflects underlying biological variation across colonies, which we specifically interested in, we retained all terms to preserve biologically meaningful contrasts. We found limited evidence of multicollinearity for the behavioural parameters (Table S3). $U_{i}^{\text{season}}\sim Ν\left(0,\sigma_{s}^{2}\right)$ was included as a random effect for season to account for inter‐annual variation in breeding success. As >90% pairs had only one observation, we did not include a random effect for pair identity, as this resulted in poor parameter estimation and model stability.

Each behavioural metric was included as a fixed effect and interacted with colony to assess potential population‐specific effects. We applied weakly informative normal priors to the fixed behavioural effects, informed by previous work on foraging strategies in albatrosses. Previous studies suggest that longer foraging trips may be associated with high breeding success due to the increased opportunity to gain food (black‐browed albatrosses: Pinaud & Weimerskirch, 2002; Weimerskirch, 1999; wandering albatrosses: Cornioley et al., 2016). In contrast, behavioural mismatches between partners, expressed as differences in mean trip duration or variability, potentially indicate reduced coordination and have been linked to lower reproductive performance (black‐browed albatrosses: Patrick & Weimerskirch, 2014; wandering albatrosses: McCully et al., 2022). Thus, the following priors were chosen:

$$\beta_{1}\sim Ν\left(-0.5,0.5\right),\beta_{2}\sim N\left(-0.25,0.5\right),\beta_{3}\sim N\left(-0.12,0.5\right)$$

The models were run with 4 chains, each consisting of 20,000 iterations, with a burn‐in of 10,000 iterations, and thinning every 10th iteration, yielding 1000 post burn‐in samples per chain. Convergence was assessed using the $\hat{R}$ statistic, with all values close to 1.00. We tested the sensitivity of our models to prior specification by comparing outputs from models fitted with weaker priors (same mean, greater variance) and non‐directional priors (mean set at 0, same variance) and found no effect on posterior distributions (Figure S3).

To assess the ecological significance of our parameter estimates, we used a Region of Practical Equivalence (ROPE) approach, which helps distinguish between statistically significant but negligible effects and those that are meaningfully large (Kruschke, 2018). The ROPE defines a range of parameter values that are considered too small to have a meaningful impact—in other words, values within this range are treated as practically equivalent to zero. For our purposes, we defined the ROPE interval as [−0.1, 0.1], reflecting biologically negligible changes in breeding success probability (~±1 percentage point at baseline success of 80%). This interval was chosen as breeding success in both species is typically very high, and so even small probability changes are likely to be meaningful. For each parameter, we calculated the proportion of the full posterior distribution falling inside the ROPE using the rope() function from the bayestestR package (Makowski et al., 2019). We interpreted parameters as having strong evidence for a meaningful effect if the 95% highest density interval (HDI) lay outside the ROPE, and weak‐to‐moderate evidence if more than 90% of the HDI fell outside.

### 2.4 Ethical approval

All fieldwork carried out at Bird Island was carried out under permit from the Government of South Georgia and the South Sandwich Islands and approved by the British Antarctic Survey's Animal Welfare Ethical Review Process and Stony Brook University's Institutional Animal Care and Use Committee (IACUC number 1473497). The field procedures and manipulations on Crozet and on Kerguelen, after approval from Comité National de la Protection de la Nature, Ethics Committee of IPEV and Comité de l'Environnement Polaire, were given permission by the ‘Préfet of Terres Australes et Antarctiques Françaises’ to Program IPEV No. 109 (PI H Weimerskirch/ C Barbraud).

## 3 RESULTS

Our analyses provided evidence that both the overall level of parental investment and the degree of coordination between the partners influenced breeding success in both black‐browed and wandering albatrosses, with several effects showing similar patterns across species at the same (or nearby, in the case of Possession Island and Kerguelen) islands (Figure 2). However, the magnitude of these effects was somewhat smaller in wandering albatrosses, and effects were more pronounced during brooding than during incubation in both species. We found consistent among‐season variation in breeding success across all models (Tables 1 and 2). This among‐season variance was greater during brooding (SD = 1.99 for black‐browed albatrosses; 1.07 for wandering albatrosses) than incubation (SD = 0.54 and 0.61 respectively), suggesting stronger annual effects later in the breeding season. We found that females took longer foraging trips on average than males during incubation, but not brooding, in both species (Figure S4).

**FIGURE 2:**
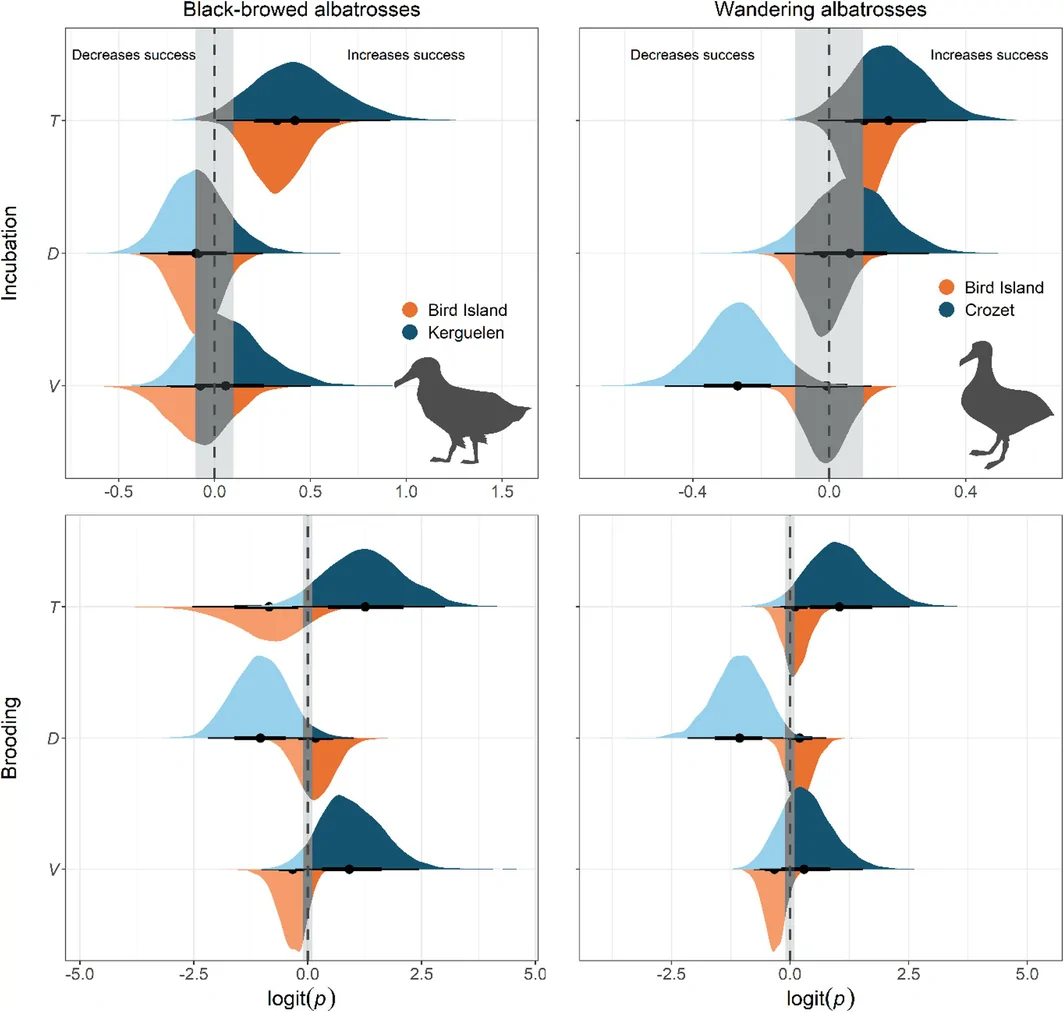
Posterior distributions (coloured ridges) and 95% credible intervals (black dot and horizontal lines) from Bayesian models examining the effects of pair‐level foraging behaviours, each measured in days, (T = pair‐level median trip duration; D = within‐pair difference in trip duration; V = within‐pair difference in trip variability) on breeding success (p) during incubation (top panels) and brooding (bottom panels). Ridges are coloured by colony (blue for Kerguelen and Crozet, orange for Bird Island). The grey vertical bar in each panel bar indicates the Region of Practical Equivalence (ROPE); effects within this region are considered negligible. The greater the proportion of the ridge extending beyond the ROPE, the stronger the evidence for a true effect. Distributions on the right of the ROPE indicate a positive effect on breeding success, and on the left indicate a negative effect.

**TABLE 1:** Parameter estimates in Bayesian binomial regression models examining the influence of pair‐level foraging metrics on breeding success in black‐browed albatrosses during incubation and brooding periods.

Parameter	Incubation			Brooding		
	Estimate ± standard error	95% CIs	Inside ROPE	Estimate ± standard error	95% CIs	Inside ROPE
Intercept	−1.41 ± 1.08	[−3.65, 0.62]	0.03	1.67 ± 1.49	[−1.1, 4.85]	0.03
Colony (Kerguelen)	3.21 ± 1.37	[0.65, 5.96]	0.00	0.06 ± 1.87	[−3.84, 3.53]	0.05
Pair‐level median trip duration	**0.33 ± 0.13**	**[0.1, 0.6]**	**0.03**	−0.91 ± 0.76	[−2.53, 0.44]	0.06
Within‐pair difference in trip duration	−0.08 ± 0.11	[−0.3, 0.13]	0.52	0.18 ± 0.41	[−0.6, 1]	0.18
Within‐pair difference in trip variability	−0.07 ± 0.16	[−0.39, 0.24]	0.43	−0.35 ± 0.31	[−1.01, 0.18]	0.16
Pair‐level median trip duration: Colony	0.1 ± 0.26	[−0.41, 0.64]	0.28	**2.19 ± 1.16**	**[0.06, 4.61]**	**0.01**
Within‐pair difference in trip duration: Colony	−0.01 ± 0.2	[−0.37, 0.41]	0.40	**−1.22 ± 0.73**	**[−2.66, 0.19]**	**0.02**
Within‐pair difference in trip variability: Colony	0.15 ± 0.25	[−0.32, 0.67]	0.28	**1.32 ± 0.77**	**[−0.08, 2.95]**	**0.02**
(1|season)	0.54 ± 0.41	[0.02, 1.54]		1.99 ± 0.87	[0.8, 4.01]	

*Note*: Estimates are reported as log odds ‘Inside ROPE’ gives the proportion of posterior samples falling within the Region of Practical Equivalence (ROPE), a range at which effects are considered minimal. Estimates where >95% of posterior samples fell outside the ROPE are highlighted in bold. ‘Bird Island’ is the reference value for the categorical predictor ‘colony’.

**TABLE 2:** Parameter estimates in Bayesian binomial regression models examining the influence of pair‐level foraging metrics on breeding success in wandering albatrosses during incubation and brooding periods.

Parameter	Incubation			Brooding		
	Estimate ± standard error	95% CIs	Inside ROPE	Estimate ± standard error	95% CIs	Inside ROPE
Intercept	0.94 ± 0.42	[0.11, 1.77]	0.02	2.84 ± 0.95	[1.07, 4.84]	0
Colony (Crozet)	0.37 ± 0.71	[−1.02, 1.75]	0.10	−0.81 ± 1.86	[−4.49, 2.87]	0.04
Pair‐level median trip duration	0.1 ± 0.06	[−0.01, 0.22]	0.48	0.13 ± 0.26	[−0.36, 0.67]	0.29
Within‐pair difference in trip duration	−0.02 ± 0.06	[−0.13, 0.1]	0.89	0.21 ± 0.26	[−0.27, 0.77]	0.24
Within‐pair difference in trip variability	−0.01 ± 0.06	[−0.13, 0.12]	0.88	−0.33 ± 0.22	[−0.76, 0.09]	0.12
Pair‐level median trip duration: Colony	0.07 ± 0.13	[−0.17, 0.33]	0.52	0.96 ± 0.76	[−0.45, 2.49]	0.05
Within‐pair difference in trip duration: Colony	0.08 ± 0.13	[−0.16, 0.33]	0.48	**−1.3 ± 0.59**	**[−2.54, −0.23]**	**0.01**
Within‐pair difference in trip variability: Colony	−0.26 ± 0.12	[−0.51, −0.03]	0.09	0.67 ± 0.6	[−0.42, 1.95]	0.07
(1|season)	0.61 ± 0.27	[0.13, 1.2]		1.07 ± 0.62	[0.09, 2.45]	

*Note*: Estimates are reported as log odds ‘Inside ROPE’ gives the proportion of posterior samples falling within the Region of Practical Equivalence (ROPE), a range at which effects are considered minimal. Estimates where >95% of posterior samples fell outside the ROPE are highlighted in bold. ‘Bird Island’ is the reference value for the categorical predictor ‘colony’.

During incubation, we found strong evidence that breeding success in black‐browed albatrosses increased with pair‐level median trip duration (T) at Bird Island, and weak evidence for an effect at Kerguelen (Table 1). The estimated effect (log odds with 95% credible intervals) at Bird Island was 0.33 [0.1–0.6] (proportion inside ROPE = 0.03). For an increase in trip duration from 2 to 5 days, this corresponded to an approximate increase in breeding success of 20 [9.4–18.07] percentage points (Figure 3a). At Kerguelen, the estimated effect was 0.1 [−0.41–0.64] (proportion inside ROPE = 0.28) corresponding to a 5 [1.09–12.4] percentage point increase in breeding success. We found no strong evidence that difference in trip duration (D), or difference in trip variability (V) affected breeding success during incubation.

**FIGURE 3:**
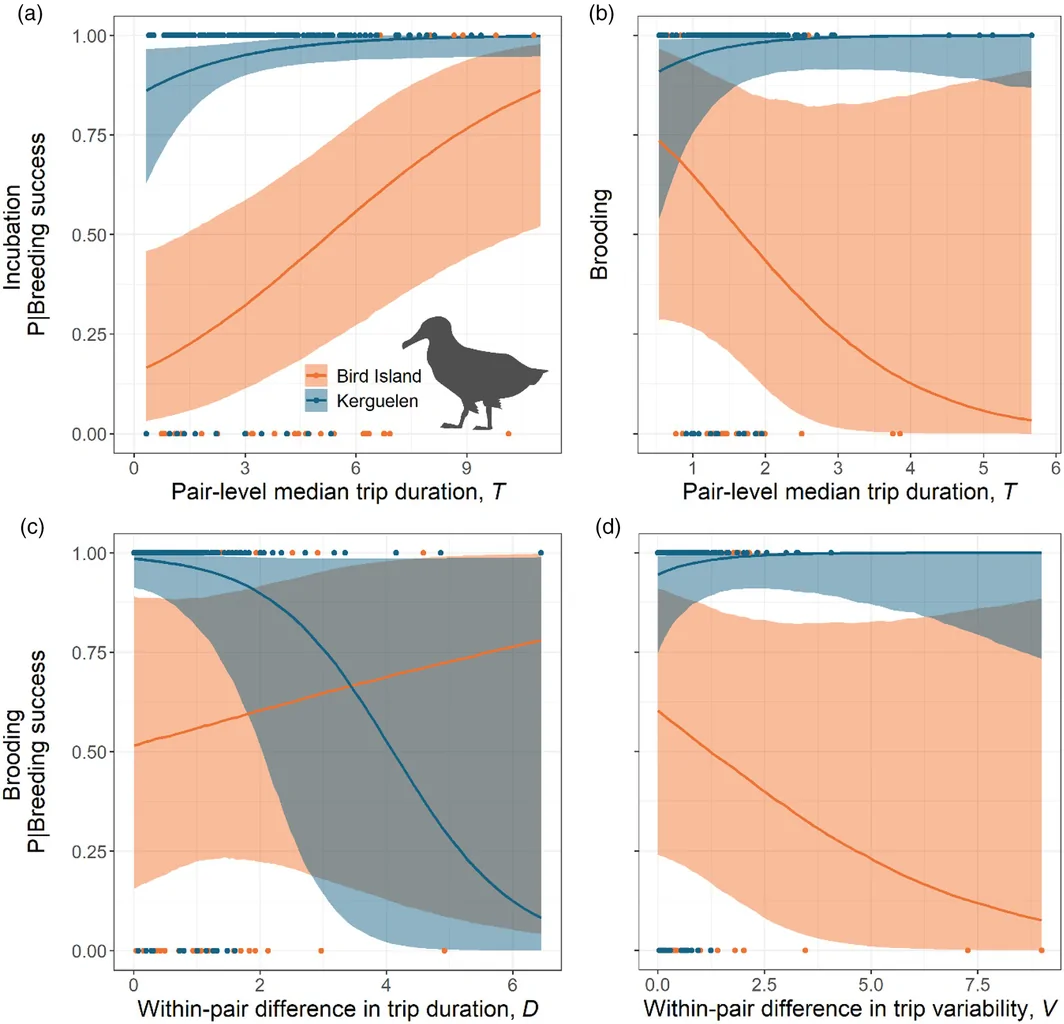
Conditional effect estimates of (a) pair‐level median trip duration, T, during incubation, and (b) pair‐level median trip duration, T, (c) within‐pair difference in trip duration, D, and (d) within‐pair difference in trip variability, V, during brooding on breeding success of black‐browed albatrosses. All behaviours are measured in days. Effects are shown separately for Bird Island (orange) and Kerguelen (blue). Solid lines represent the estimated mean effect, and the shaded areas denote the 95% credible interval. Data points correspond to individual pair estimates. Note that some estimates may extend outside the range of observed data due to extrapolation and should thus be interpreted with caution.

During brooding, we found moderate evidence for a positive effect of pair‐level median trip duration (T) on breeding success at Kerguelen, where the environment is less variable, but not Bird Island, where the environment is more variable, (interaction estimate = 2.19 [0.06–4.61], proportion in ROPE = 0.01; Table 1; Figure 3b). This equates to an approximate increase in breeding success of 7 [0.04–28.9] percentage points for an increase in trip duration from 1 to 2 days (Figure 3b). We also found strong evidence that, in contrast, greater within‐pair differences in trip duration (D) reduced breeding success at Kerguelen (interaction estimate = −1.22 [−2.66–0.19], proportion in ROPE = 0.02; Table 1), equating to a 14 [0.09–47.65] percentage point decline for an increase in duration differences from 0 to 2 days (Figure 3c). Finally, we found strong evidence that greater within‐pair differences in trip variability (V) increased breeding success at Kerguelen (interaction estimate = 1.32 [−0.08–2.95], proportion in ROPE = 0.02; Table 1), equating to a 8 [0.04–32.02] percentage point increase for an increase in duration differences from 0 to 2 days (Figure 3d). We found negligible evidence for effects of any behaviour at Bird Island.

For wandering albatrosses, we found negligible evidence that pair‐level foraging metrics during incubation or brooding influenced breeding success at Bird Island (Table 2). At Crozet, during incubation, we found weak evidence for a negative effect of within‐pair differences in trip variability (V) on breeding success (interaction estimate = −0.26 [−0.51–0.03], proportion inside ROPE = 0.09; Table 2), corresponding to an approximate decline in breeding success of −9 [−16.86–3.15] percentage points for an increase in difference from 0 to 3 days. There was negligible evidence for effects of the remaining two behaviours (T and D).

During brooding at Crozet, we found strong evidence that within‐pair differences in trip duration (D) reduced breeding success by approximately 16 [0.36–53.7] percentage points (interaction estimate = −1.3 [−2.54–0.23], proportion inside ROPE = 0.01; Table 2; Figure 4). We found weak evidence for positive effects of median trip duration (T; interaction estimate = 0.67 [−0.42–1.95], proportion inside ROPE = 0.07) and within‐pair differences in variability, (V; interaction estimate = 0.67 [−0.42–1.95], proportion inside ROPE = 0.07) on breeding success.

**FIGURE 4:**
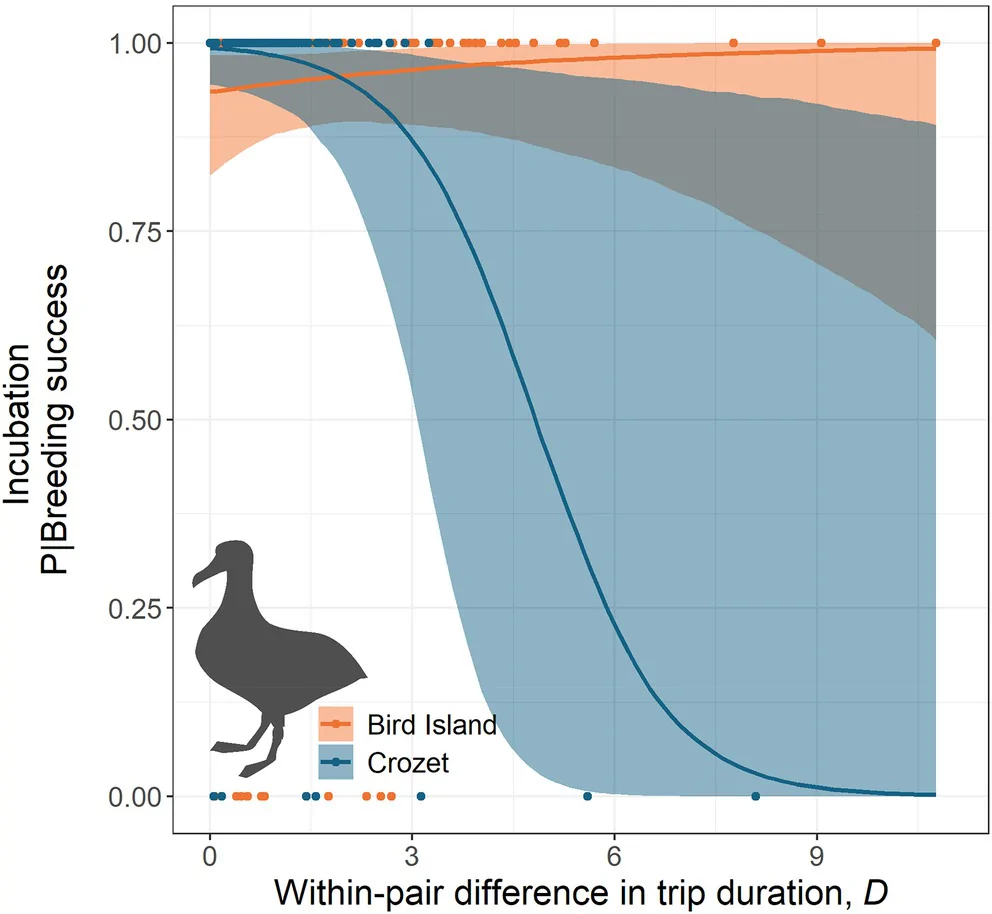
Conditional effect estimates of within‐pair difference in trip duration, D, during brooding on breeding success of wandering albatrosses. All behaviours are measured in days. Effects are shown separately for Bird Island (orange) and Crozet (blue). Solid lines represent the estimated mean effect, and the shaded areas denote the 95% credible interval. Data points correspond to individual pair estimates.

## 4 DISCUSSION

Albatrosses are renowned for their wide‐ranging foraging capabilities (Weimerskirch et al., 1988), which, coupled with their physiological capacity to endure prolonged fasting at the nest (Weimerskirch, 1999; Weimerskirch et al., 1988), should enable high levels of flexibility in their behaviour. However, as biparental species, successful breeding (defined here as offspring survival to fledging) depends not just on the behaviour of the individual but also that of its partner. How parents navigate this constraint is dictated both by their physiology and the environmental conditions at their breeding grounds. We found that, for both black‐browed and wandering albatrosses, the relative investment of the parents, measured as trip duration, generally increased breeding success, and that coordination, measured as more similar trip durations between the pair members, had a positive impact on breeding success. Unexpectedly, we found that these effects were less important at Bird Island, where environments are reported to be more variable (Murphy et al., 2007; Nevoux et al., 2010; Trathan & Murphy, 2002). This may suggest that coordination can enhance reproductive success, but its importance relative to individual flexibility diminishes as the environment becomes more unpredictable.

In stable and predictable environments, the ability to coordinate with the partner and to take prolonged foraging trips may allow parents to partition their effort across the pair and hence increase future reproductive success (Patrick et al., 2020). This may explain why longer trips during incubation and brooding, and smaller within‐pair differences in trip duration (during brooding) were associated with higher breeding success at Kerguelen and Crozet. This is probably especially beneficial to black‐browed albatrosses at Kerguelen, where adult survival is lower and so each breeding attempt holds greater value (Nevoux et al., 2010). Conversely, at Bird Island we detected few effects of relative investment and coordination on reproductive success in both species, suggesting that fluctuating foraging conditions override the advantages of partner coordination, a finding that did not match our initial prediction that environmental and reproductive variability would select for greater importance of coordination.

It remains an open question as to how the environment is expected to shape the prevalence and extent to which parents should provide coordinated care (Bonsall & Klug, 2011). Theoretical predictions and empirical evidence variously suggest that the value of parental care is higher in stochastic environments (Vincze et al., 2017), selects for self‐serving strategies (Johnstone & Hinde, 2006; McNamara et al., 1999) or has no effect on provisioning in biparental species (Remeš et al., 2015). For long‐lived species, where a single breeding attempt represents a small proportion of potential lifetime reproductive output, unpredictable conditions may erode the value of parental coordination, as it can be more important for individuals to respond flexibly to their immediate environment. As a result, strict coordination between the parents may not be an optimal strategy: even well‐coordinated pairs may struggle if foraging conditions are poor (e.g. Xavier et al., 2013), while, conversely, poorly coordinated pairs might still be successful if foraging conditions are very good. Furthermore, the benefits of coordination might not be uniform across all individuals: pairs composed of high‐quality parents may be better able to maintain coordination without compromising individual condition, whereas lower‐quality individuals might lack the capacity to both coordinate and meet their own energetic needs. This has been proposed as an explanation for the low levels of coordination observed in little penguins *Eudyptula minor*, despite such coordination being associated with higher breeding success (Saraux et al., 2011). At the extreme, the need for individuals to respond flexibly and dynamically to environmental variation could shift selection away from cooperative strategies and toward more individualistic behaviours that place greater parental burden on the partner. However, this strategy may not be sustainable in the long term, as an overburdened partner may be more likely to divorce (Choudhury, 1995; Husak & Moore, 2008). For albatrosses, retaining a partner over multiple years is thought to be beneficial (Bried et al., 2003), and remating is associated with significant reproductive costs (Jouventin et al., 1999; Ventura et al., 2021), meaning such a shift could ultimately reduce fitness rather than enhance it.

At Bird Island, the only positive effect detected was that of longer incubation trips on breeding success in black‐browed albatrosses. Since there is no requirement to forage for the chick during incubation, longer trips may allow individuals to gain more reserves, helping them sustain longer incubation and brooding stints (Pinaud & Weimerskirch, 2002; Weimerskirch, 1999). This effect was observed at both black‐browed albatross colonies (though the evidence for Kerguelen was relatively weak), highlighting the general importance of recovering or increasing body reserves during incubation. However, during brooding, the importance of trip duration varied: at Kerguelen, longer trips improved breeding success, whereas at Bird Island, they had little effect. The pattern at Kerguelen suggests a threshold duration required to secure sufficient food for chicks (Phillips et al., 2009; Weimerskirch, Cherel, et al., 1997; Weimerskirch, Mougey, & Hindermeyer, 1997), which may remain consistent across seasons due to relatively stable conditions. For wandering albatrosses, only weak evidence for effects of trip duration during brooding on breeding success were detected, and only at Crozet. However, as for black‐browed albatrosses, the direction of this effect was positive. Wandering albatrosses rely on long‐distance foraging, travelling thousands of kilometres to exploit widely dispersed prey (Phillips et al., 2009; Weimerskirch, 1995; Weimerskirch et al., 1993; Xavier et al., 2004). Their large body size allows them to endure prolonged fasting while incubating, enabling the partner to travel long distances to replenish reserves at productive foraging sites (Cornioley et al., 2016), and potentially explaining why incubation trip duration does not influence breeding success. However, brooding is an inherently more energetically demanding period, as parents must balance the conflicting needs of themselves, their partner and their chick (Bevan et al., 1995; Ricklefs, 1983). This trade‐off between self‐maintenance and chick provisioning may therefore make foraging trip duration more consequential at this stage. Thus, extended absences during brooding—that is longer trips—may indicate that birds prioritise their own body reserves when feeding conditions allow the chick to be provisioned effectively.

At both Kerguelen and Crozet, larger within‐pair differences in trip duration reduced breeding success, suggesting that consistency within pairs is beneficial. Although the wide confidence intervals mean these results must be treated with caution, behavioural incompatibility has been linked to lower reproductive success in several species (Both et al., 2005; Ihle et al., 2015; McCully et al., 2023; Schuett et al., 2011; Spoon et al., 2006), supporting the idea that similarity in partners' behaviour may be advantageous. Interestingly, for black‐browed albatrosses we observed a trend for greater differences in trip variability to be associated with higher breeding success, though the effect was relatively small. The reason for this is unclear, but one possibility is that variation in individual foraging strategies may allow parents to complement one another's behaviour even under variable conditions. For wandering albatrosses, only negative effects of differences in trip duration were found. Wandering albatrosses are highly resilient to environmental perturbations: Their large size allows them to exploit a range of wind conditions for efficient, low‐cost travel over vast distances, enabling greater energetic investment in reproduction, and they can endure prolonged fasting while incubating (Weimerskirch et al., 1993). Their ability to cope with short‐term environmental changes (Gillies et al., 2024) suggests they should be well‐equipped to buffer effects of environmental variation on breeding success. While breeding failure is more common during brooding than incubation for black‐browed albatrosses at Kerguelen (Table S4), for wandering albatrosses at Crozet, breeding failure is more common during incubation (Weimerskirch et al., 2012). Despite this, our results suggest that reduced coordination during brooding may also undermine success. Notably, declines in breeding success were only observed when these differences were large (>5 days), suggesting that parents can tolerate moderate asynchrony but struggle with extreme mismatches. These mismatches may reflect high energetic stress or imbalance in parental investment, which may be harder to tolerate during the chick‐provisioning stage.

Our results provide some insight into the timescale at which coordination may be important. Theoretical work predicts that parents should act to minimise the accumulation of energetic and survival costs incurred during breeding (Williams, 1966). However, in biparentally caring species that form long‐term monogamous pair bonds, this must be balanced against the need to maintain the partner's condition, as future reproductive success may depend, at least in part, on the continued viability of the pair (Griffith, 2019). Parents may achieve this by adjusting to the investment of their partner over a short timescale (e.g. trip by trip), or over the entire breeding period (Harrison et al., 2009; Johnstone et al., 2014; Johnstone & Hinde, 2006), both of which have empirical support (Baldan & van Loon, 2022; Gillies, Syposz, et al., 2021; Kavelaars et al., 2021). For black‐browed albatrosses at Kerguelen and wandering albatrosses at Crozet, pairs with more similar median trip durations had higher breeding success, suggesting that responding to the partner on a trip‐by‐trip basis, rather than securing an overall balance in investment, is key to successful breeding. These effects were strongest during brooding, which may reflect a compounding effect of long trip durations on chick provisioning if body condition of one of the parents is compromised. Consistent foraging trip durations are believe to be highly beneficial during brooding, as chicks do best when fasting periods between meals are short and predictable (Weimerskirch et al., 2000). If one parent is consistently taking shorter trips, this may lead to prolonged or intermittent fasting for the chick and therefore suboptimal provisioning, even if that parent compensates with more frequent trips.

Our results demonstrate that the relationship between parental behaviour, specifically coordination between breeding pairs, and breeding success is associated with environmental context. Albatrosses are highly reliant on dynamic and unpredictable marine resources to forage and provide for their offspring. Yet perhaps unintuitively, coordination appeared to be more valuable to birds breeding in stable environments. At Bird Island, differences in coordination may no longer be the primary determinant of success: Instead, external factors such as environmental change (Darby et al., 2024), prey availability (Atkinson et al., 2019), or fisheries interactions (for wandering albatrosses; Xavier et al., 2004) may counter the benefits of coordination, favouring individuals that can flexibly adjust their behaviour rather than prioritising synchronicity with their partner. In such conditions, selection may act against rigid coordination.

However, while our comparative framework allowed us to contrast colonies experiencing differing levels of environmental variation, our results should be interpreted with nuance. First, it is important to note that many of our estimates were associated with wide confidence intervals and small effect sizes, and while this reflects the inherent complexity of these long‐lived systems, they should be interpreted with caution. Furthermore, although environmental variability is a plausible driver of these divergent strategies, our models identified associations rather than direct causation. Other site‐specific factors, such as population demography and social dynamics, may also contribute to the observed inter‐colony differences. Consequently, while we identified distinct behavioural signatures at each site, we cannot definitively isolate environmental variation as the sole driver. To untangle these complex dynamics, future research exploring the relevance of environmental variation at different temporal scales is needed. In general, adopting a pair‐level perspective that quantifies how individuals differentially invest in themselves and their offspring across different ecological and environmental contexts will give essential insight into the evolution of biparental care, particularly in a world subject to increasing environmental variability.

## AUTHOR CONTRIBUTIONS

Natasha Gillies, Richard A. Phillips, Henri Weimerskirch, Jonathan R. Potts, Denis Réale, Alastair Wilson and Samantha C. Patrick conceived the ideas and designed the study. Natasha Gillies and Jonathan R. Potts conducted the analyses. Frédéric Angelier, Christophe Barbraud, Ashley Bennison, Karine Delord, Prescillia Lemesle, Samuel Peroteau, Andrew G. Wood and José C. Xavier carried out data collection and management. All authors contributed to the drafting and writing of the manuscript.

## CONFLICT OF INTEREST STATEMENT

The authors declare no conflicts of interest.

## ETHICS STATEMENT

All fieldwork carried out at Bird Island was approved by the British Antarctic Survey Animal Welfare and Ethical Review Body and was carried out under permit from the Government of South Georgia and the South Sandwich Islands. The field procedures and manipulations on Crozet and on Kerguelen, after approval from Comité National de la Protection de la Nature, Ethics Committee of IPEV and Comité de l'Environnement Polaire, were given permission by the ‘Préfet of Terres Australes et Antarctiques Françaises’ to Program IPEV No. 109 (PI H Weimerskirch/ C Barbraud).

## STATEMENT ON INCLUSION

Our study involves authors from various countries, including individuals affiliated with the country holding administrative control over the region where the data were gathered. All authors were involved from the outset in shaping the research and study design to encompass the broad range of perspectives they bring.

## Supporting information


**Table S1.** Sample sizes used in the study. ‘Observations’ denote the summarized breeding behaviours recorded for each pair throughout the breeding season, with each observation representing the collective behaviours of one pair. Parentheses contain the number of pairs/observations for pairs where sex was known. BBAL, black‐browed albatross; WAAL, wandering albatross.
**Table S2.** Mean and spatial standard deviation of per‐cell temporal standard deviation (SD) and coefficient of variation (CV) of sea‐surface temperature (SST) within each colony's foraging range. SD of SST indicates variability over time; CV of SST indicates variability relative to mean SST over time. N cells indicates the number of cells used in the summary calculations.
**Figure S1.** Spatial distribution of relative temporal sea‐surface temperature variability within the core foraging ranges of black‐browed albatrosses at (A) Bird Island and (B) Kerguelen and wandering albatrosses at (C) Bird Island and (D) Crozet. Colours represent the coefficient of variation (CV) of weekly SST within each 0.25° grid cell over the breeding season. Warmer colours indicate greater temporal variability relative to the local mean SST, while cooler colours indicate more stable thermal conditions. Black triangles indicate colony locations.
**Figure S2.** Schematic illustrating the behavioural parameters used in the analysis. Purple and yellow represent two individuals within a pair. Coloured horizontal and vertical lines indicate the mean and standard deviation, respectively. Each set of coloured points represents sequential foraging trips by one individual, with each pair shown in a single panel. Panels illustrate scenarios in which individuals within a pair have (A) similar mean trip durations and similar variability; (B) different mean durations and similar variability; (C) similar mean durations and different variability; and (D) different mean durations and different variability.
**Table S3.** Variance Inflation Factors (VIFs) for fixed effects in the breeding success models used in the analyses. Values <5 indicate low multicollinearity. VIF estimates include 95% confidence intervals. BBAL, black‐browed albatross; WAAL, wandering albatross.
**Figure S3.** Comparison of posterior estimates across different prior specifications: the reference priors used in the main analysis, weaker priors with the same directionality but greater variance, and non‐directional priors with the same variance but centred on zero. Results are shown for black‐browed albatrosses during (A) incubation and (B) brooding, and wandering albatrosses during (C) incubation and (D) brooding. Panels show posterior distributions (coloured ridges) and 95% credible intervals (black dot and horizontal lines) from Bayesian models examining the effects of pair‐level foraging behaviours, each measured in days, (T = pair‐level median trip duration; D = within‐pair difference in trip duration; V = within‐pair difference in trip variability) on breeding success (p). Ridges are coloured by colony (blue for Kerguelen and Crozet, orange for Bird Island). The grey vertical bar in each panel bar indicates the Region of Practical Equivalence (ROPE); effects within this region are considered negligible. The greater the proportion of the ridge extending beyond the ROPE, the stronger the evidence for a true effect. Distributions on the right of the ROPE indicate a positive effect on breeding success, and on the left indicate a negative effect.
**Figure S4.** Distribution of foraging trip duration across breeding phases, by sex. Boxplots show trip duration (days) by breeding phase, with data shown separately for females (purple) and males (yellow). Each species‐colony combination is shown in a separate panel (BBAL, black‐browed albatross; WAAL, wandering albatross).
**Table S4.** Summary of breeding success across each species and colony, expressed as a percentage of the total number of observations.
**Figure S5.** Residual diagnostics for Bayesian Generalized Linear Mixed Models evaluating the effects of coordination metrics on breeding success. Panels are organised in rows by species and life stage (top to bottom): black‐browed albatross (BBAL) and wandering albatross (WAAL) during incubation and brooding. Each row displays randomized quantile residuals (DHARMa package) including: QQ plots to assess distribution and outliers (leftmost panels), residuals vs. categorical predictors to verify homogeneity of variance (centre panels), and residuals vs. rank‐transformed trip duration to check for non‐linearity (rightmost panels). Across all models, Kolmogorov–Smirnov, dispersion, and outlier tests confirm that the models are well‐specified and free from significant overdispersion. While minor heteroscedasticity and quantile deviations were detected for WAAL during brooding (bottom row), likely due to the small number of failures during chick provisioning, these were considered negligible as they did not affect the global model stability or the interpretation of the main effects.

## Data Availability

All data supporting the results as well as associated scripts to conduct the analyses can be accessed on Zenodo at https://doi.org/10.5281/zenodo.15490196 (Gillies, 2026b). Additional raw activity, tracking, and breeding success data are publicly accessible by location and species as follows. For Kerguelen and Crozet, data for black‐browed and wandering albatrosses respectively are hosted at https://doi.org/10.48579/PRO/77GAOX (Gillies, 2026a). For Bird Island black‐browed albatrosses, raw activity data are available at https://doi.org/10.5285/3bcd59f9‐1068‐4474‐bca9‐c8ca9fe0caed (Phillips, Wakefield, Thorne, & Wood, 2026), tracking data at https://doi.org/10.5285/249ce261‐284c‐4e26‐bcab‐1a57cee7de5e (Phillips, Wakefield, & Thorne, 2026) and breeding success data at https://doi.org/10.5285/8e303c28‐c96a‐49e7‐951d‐2d69c0b6f032 (Phillips, Bennison, & Wood, 2026b). For Bird Island wandering albatrosses raw activity data are available at: https://doi.org/10.5285/8e303c28‐c96a‐49e7‐951d‐2d69c0b6f032 (Phillips, Carneiro, Catry, et al., 2026); tracking data at: https://doi.org/10.5285/1a916a0a‐adcd‐4565‐9de9‐b535000fd6f8 (Phillips, Carneiro, Catry, et al., 2026) and breeding success at https://doi.org/10.5285/1510b98d‐2807‐4d48‐9156‐43136ce8a3c3 (Phillips, Bennison, & Wood, 2026a).
