# Supplementary material for: Contrasting associations between parental coordination and breeding success in albatrosses across differing environmental contexts
Source: J Anim Ecol. 2026 Sep 15;95(11):2306–21. doi: 10.1111/1365-2656.70350 (PMC13731832; doi:10.1111/1365-2656.70350)
Supplement: Supplementary file 1 [file JANE-95-2306-s001.docx]

**Supplementary Materials for: *Contrasting associations between parental coordination and breeding success in albatrosses across differing environmental contexts***

**This document contains:**

[Table S1	2](#_Toc224641447)

[Comparing environmental variation	3](#_Toc224641448)

[Table S2.	4](#_Toc224641449)

[Figure S1.	5](#_Toc224641450)

[Figure S2.	6](#_Toc224641451)

[Table S3.	7](#_Toc224641452)

[Sensitivity of model outputs to prior specification	8](#_Toc224641453)

[Figure S3.	10](#_Toc224641454)

[Sex differences in investment	11](#_Toc224641455)

[Figure S4	12](#_Toc224641456)

[Table S4.	13](#_Toc224641457)

[Figure S5	15](#_Toc224641458)

[References	16](#_Toc224641459)

Table S1. Sample sizes used in the study. ‘Observations’ denote the summarized breeding behaviours recorded for each pair throughout the breeding season, with each observation representing the collective behaviours of one pair. Parentheses contain the number of pairs/observations for pairs where sex was known. BBAL = black-browed albatross; WAAL = wandering albatross.

| **Species** | **Colony** | **Phase** | **Pairs** | **Observations** | **Seasons** |
| --- | --- | --- | --- | --- | --- |
| BBAL | Bird Island | Incubation | 46 (37) | 50 (39) | 2004, 2010, 2020, 2021, 2022, 2023 |
|  |  | Brooding | 126 (120) | 210 (203) | 2007, 2008, 2009, 2010, 2011, 2012, 2013, 2014 |
|  | Kerguelen | Incubation | 198 (198) | 265 (265) | 1999, 2001, 2004, 2005, 2006, 2007, 2008, 2009, 2010, 2011, 2012, 2019, 2020, 2021, 2022, 2023 |
|  |  | Brooding | 152 (152) | 201 (201) | 2007, 2008, 2009, 2010, 2011, 2012, 2013, 2014, 2016, 2019 |
| WAAL | Bird Island | Incubation | 40 (35) | 46 (41) | 2002, 2003, 2008, 2009, 2010, 2015, 2020, 2021, 2022 |
|  |  | Brooding | 141 (135) | 246 (237) | 2008, 2009, 2010, 2011, 2012, 2013, 2014, 2015 |
|  | Crozet | Incubation | 135 (135) | 152 (152) | 1991, 1999, 2003, 2004, 2005, 2006, 2007, 2008, 2009, 2010, 2011, 2018, 2019, 2020, 2021, 2022, 2023 |
|  |  | Brooding | 69 (69) | 77 (77) | 2008, 2009, 2010, 2011, 2012, 2013 |

Comparing environmental variation

To quantify spatio-temporal environmental variability, we examined variation in sea surface temperature (SST) over time at each colony. SST is a widely used metric in seabird ecology as it reflects conditions that influence the distribution and abundance of prey, and has wide-ranging effects on a number of seabird species (Guinet et al. 1998; Irons et al. 2008; Devney et al. 2009; Wakefield et al. 2011; Carroll et al. 2015; Pardo et al. 2017; Gillies et al. 2024).

We obtained SST data from the NOAA Optimum Interpolation Sea Surface Temperature Version 2 High Resolution Dataset (Huang et al. 2021; accessed 06/03/2026), which provides a global blended analysis of SST at 0.25° spatial resolution. We extracted weekly SST estimates during the breeding season from within the core foraging range of each colony, defined as the 80% utilization distribution (UD) derived from GPS tracking data. To quantify environmental variability within these spatial domains, we calculated two metrics for each grid cell: the standard deviation (SD) of SST through time, providing an absolute measure of temporal environmental variability at that location, and the coefficient of variation (CV; SD divided by the mean SST within the cell) to quantify relative temporal variability, by accounting for spatial differences in mean temperature.

We used linear models to compare per-cell SD and CV values between colonies, treating each grid cell as the sampling unit. At the colony level, we summarised the mean of per-cell SD and CV values to characterise overall temporal variability experienced by each population, and the standard deviation of these values to quantify spatial heterogeneity in temporal variability within each colony.

Summarised values of environmental variability indicated that Bird Island had higher absolute temporal variability, greater spatial heterogeneity, and higher relative variability compared to both Kerguelen and Crozet (Table S2, Figure S1).

Analyses of per-cell SD and CV values showed that absolute SST was more variable around Bird Island over time (SD SST: t = 67.95, df = 11789, p < 0.0001) and that this pattern held when considering variability relative to local mean SST (CV SST: t = 27.45, df = 10032, p < 0.0001).

The same patterns were observed when comparing variability between Bird Island and Crozet for the foraging range of wandering albatrosses, where again Bird Island was more variable in absolute SST over time (SD SST: t = 61.11, df = 23294, p < 0.0001) and when considering relative variability (CV SST: t = 94.27, df = 21537, p = < 0.001).

Table S2. Mean and spatial standard deviation of per-cell temporal standard deviation (SD) and coefficient of variation (CV) of sea-surface temperature (SST) within each colony’s foraging range. SD of SST indicates variability over time; CV of SST indicates variability relative to mean SST over time. N cells indicates the number of cells used in the summary calculations.

| **Species** | **Colony** | **Mean(SD SST)** | **Standard deviation (SD SST)** | **Mean(CV SST)** | **Standard deviation (CV SST)** | **N cells** |
| --- | --- | --- | --- | --- | --- | --- |
| BBAL | Bird Island | 1.07 | 0.13 | 0.44 | 0.17 | 1974 |
| BBAL | Kerguelen | 0.79 | 0.2 | 0.18 | 0.04 | 1520 |
| WAAL | Bird Island | 1.84 | 0.6 | 0.36 | 0.26 | 13025 |
| WAAL | Crozet | 1.45 | 0.37 | 0.14 | 0.06 | 10271 |

Figure S1. Spatial distribution of relative temporal sea-surface temperature variability within the core foraging ranges of black-browed albatrosses at (A) Bird Island and (B) Kerguelen and wandering albatrosses at (C) Bird Island and (D) Crozet. Colours represent the coefficient of variation (CV) of weekly SST within each 0.25° grid cell over the breeding season. Warmer colours indicate greater temporal variability relative to the local mean SST, while cooler colours indicate more stable thermal conditions. Black triangles indicate colony locations.

Figure S2. Schematic illustrating the behavioural parameters used in the analysis. Purple and yellow represent two individuals within a pair. Coloured horizontal and vertical lines indicate the mean and standard deviation, respectively. Each set of coloured points represents sequential foraging trips by one individual, with each pair shown in a single panel. Panels illustrate scenarios in which individuals within a pair have (A) similar mean trip durations and similar variability; (B) different mean durations and similar variability; (C) similar mean durations and different variability; and (D) different mean durations and different variability.

| **Species** | **Phase** | **Term** | **VIF** |
| --- | --- | --- | --- |
| BBAL | Incubation | Colony | 3.69 [3.05, 4.53] |
|  |  | Pair-level median trip duration | 1.58 [1.38, 1.89] |
|  |  | Within-pair difference in trip duration | 1.69 [1.46, 2.02] |
|  |  | Within-pair difference in trip variability | 1.87 [1.6, 2.24] |
|  | Brooding | Colony | 3.93 [3.28, 4.77] |
|  |  | Pair-level median trip duration | 2.33 [1.99, 2.79] |
|  |  | Within-pair difference in trip duration | 2.18 [1.87, 2.61] |
|  |  | Within-pair difference in trip variability | 1.52 [1.34, 1.79] |
| WAAL | Incubation | Colony | 4.13 [3.55, 4.85] |
|  |  | Pair-level median trip duration | 1.39 [1.26, 1.58] |
|  |  | Within-pair difference in trip duration | 1.33 [1.21, 1.52] |
|  |  | Within-pair difference in trip variability | 1.42 [1.29, 1.63] |
|  | Brooding | **Colony** | **5.72 [4.67, 7.08]** |
|  |  | Pair-level median trip duration | 1.17 [1.07, 1.42] |
|  |  | Within-pair difference in trip duration | 1.72 [1.49, 2.06] |
|  |  | Within-pair difference in trip variability | 1.6 [1.39, 1.91] |

Table S3. Variance Inflation Factors (VIFs) for fixed effects in the breeding success models used in the analyses. Values < 5 indicate low multicollinearity. VIF estimates include 95% confidence intervals. BBAL = black-browed albatross; WAAL = wandering albatross.

# Sensitivity of model outputs to prior specification

We assessed sensitivity to prior specification by refitting each model under two alternative prior scenarios: (i) weaker directional priors with increased variance, and (ii) non-directional priors centred at zero for all behavioural effects. Posterior estimates were compared across models to assess consistency in effect direction, magnitude, and uncertainty (Figure S3). Results were considered robust to prior specification if posterior effect direction and qualitative inference (credible interval overlap with zero) were consistent across all prior scenarios. We found consistent posterior estimates across the priors used.

Figure S3. Comparison of posterior estimates across different prior specifications: the reference priors used in the main analysis, weaker priors with the same directionality but greater variance, and non-directional priors with the same variance but centred on zero. Results are shown for black-browed albatrosses during (A) incubation and (B) brooding, and wandering albatrosses during (C) incubation and (D) brooding. Panels show posterior distributions (coloured ridges) and 95% credible intervals (black dot and horizontal lines) from Bayesian models examining the effects of pair-level foraging behaviours, each measured in days, ($\boldsymbol{T}$ = pair-level median trip duration; $\boldsymbol{D}$ = within-pair difference in trip duration; $\boldsymbol{V}$ = within-pair difference in trip variability) on breeding success ($\boldsymbol{p}$). Ridges are coloured by colony (blue for Kerguelen and Crozet, orange for Bird Island). The grey vertical bar in each panel bar indicates the Region of Practical Equivalence (ROPE); effects within this region are considered negligible. The greater the proportion of the ridge extending beyond the ROPE, the stronger the evidence for a true effect. Distributions on the right of the ROPE indicate a positive effect on breeding success, and on the left indicate a negative effect.

# Sex differences in investment

We examined whether there was any tendency for females and males of each species to take foraging trips of different durations. For each species, we fitted a linear mixed-effects model with foraging trip duration (days) as the response, sex and breeding phase as interacting fixed effects, and colony as an additive fixed effect. We included a random intercept for individual (ring ID) to control for repeated measures. Models were fitted using the R package lme4 (Bates et al. 2015).

As reported in previous studies (Patrick and Weimerskirch 2014; McCully et al. 2022), we found that females took longer foraging trips than males during incubation, but not brooding, in both species. During incubation, we found that females took longer trips on average than males (Figure S4). For black-browed albatrosses, trips by females lasted on average 5.31 [5.1, 5.52] days versus 3.89 [3.68, 4.09] days for those by males (t = -10.4, p < 0.0001). For wandering albatrosses, trips by females lasted 6.77 [6.54, 7] days and those by males lasted 5.48 [5.26, 5.71] days (t = -5.29, p < 0.001).

During brooding, however, trips durations of males and females were similar in both species. In black-browed albatrosses, females took trips lasting a mean of 2.5 [2.3, 2.71] days, and males took trips lasting a mean of 2.44 [2.24, 2.64] days (t = -0.45, p = 0.65). Similarly, mean trip durations for female wandering albatrosses lasted 3.38 [3.03, 3.74] days, and for males lasted 3.53 [3.18, 3.88] days (t = 0.59, p = 0.56).

Figure S4. Distribution of foraging trip duration across breeding phases, by sex. Boxplots show trip duration (days) by breeding phase, with data shown separately for females (purple) and males (yellow). Each species-colony combination is shown in a separate panel (BBAL = black-browed albatross; WAAL = wandering albatross).

Table S4.  Summary of breeding success across each species and colony, expressed as a percentage of the total number of observations.

| **Species** | **Colony** | **N** | **Egg failed** | **Chick failed** | **Failure stage unknown** | **Successful** |
| --- | --- | --- | --- | --- | --- | --- |
| BBAL | Bird Island | 103 | 2.91 | 52.43 | 0.97 | 43.69 |
|  | Kerguelen | 459 | 1.53 | 3.27 | 0.22 | 94.77 |
| WAAL | Bird Island | 418 | 7.66 | 7.42 | 1.67 | 83.25 |
|  | Crozet | 280 | 4.29 | 2.50 | 6.07 | 87.14 |

Figure S5. Residual diagnostics for Bayesian Generalized Linear Mixed Models evaluating the effects of coordination metrics on breeding success. Panels are organised in rows by species and life stage (top to bottom): black-browed albatross (BBAL) and wandering albatross (WAAL) during incubation and brooding. Each row displays randomized quantile residuals (DHARMa package) including: QQ plots to assess distribution and outliers (leftmost panels), residuals vs. categorical predictors to verify homogeneity of variance (centre panels), and residuals vs. rank-transformed trip duration to check for non-linearity (rightmost panels). Across all models, Kolmogorov-Smirnov, dispersion, and outlier tests confirm that the models are well-specified and free from significant overdispersion. While minor heteroscedasticity and quantile deviations were detected for WAAL during brooding (bottom row), likely due to the small number of failures during chick-provisioning, these were considered negligible as they did not affect the global model stability or the interpretation of the main effects.

# References

Bates D, Mächler M, Bolker B, Walker S. 2015. Fitting Linear Mixed-Effects Models Using lme4. Journal of Statistical Software. 67(1):1–48. https://doi.org/10.18637/jss.v067.i01

Carroll MJ et al. 2015. Effects of sea temperature and stratification changes on seabird breeding success. Climate Research. 66:75–89. https://doi.org/10.3354/cr01332

Devney CA, Short M, Congdon BC. 2009. Sensitivity of tropical seabirds to El Niño precursors. Ecology. 90(5):1175–1183. https://doi.org/10.1890/08-0634.1

Gillies N et al. 2024. Plastic Behaviour Buffers Climate Variability in the Wandering Albatross. Ecology and Evolution. 14(12):e70631. https://doi.org/10.1002/ece3.70631

Guinet C et al. 1998. Effects of warm sea-surface temperature anomalies on the blue petrel at the Kerguelen Islands. Proceedings of the Royal Society B: Biological Sciences. 265(1400):1001–1006. https://doi.org/10.1098/rspb.1998.0390

Hartig F, Lohse L, leite M de S. 2024. DHARMa: Residual Diagnostics for Hierarchical (Multi-Level / Mixed) Regression Models. [accessed 2026 Mar 6]. https://cran.r-project.org/web/packages/DHARMa/index.html

Huang B et al. 2021. Improvements of the Daily Optimum Interpolation Sea Surface Temperature (DOISST) Version 2.1. Journal of Climate. 34(8):2923–2939. https://doi.org/10.1175/JCLI-D-20-0166.1

Irons DB et al. 2008. Fluctuations in circumpolar seabird populations linked to climate oscillations. Global Change Biology. 14(7):1455–1463. https://doi.org/10.1111/j.1365-2486.2008.01581.x

McCully FR et al. 2022. Partner intrinsic characteristics influence foraging trip duration, but not coordination of care in wandering albatrosses Diomedea exulans. Ecology and Evolution. 12(12). https://doi.org/10.1002/ece3.9621

Pardo D, Jenouvrier S, Weimerskirch H, Barbraud C. 2017. Effect of extreme sea surface temperature events on the demography of an age-structured albatross population. Philos Trans R Soc Lond B Biol Sci. 372(1723):20160143. https://doi.org/10.1098/rstb.2016.0143

Patrick SC, Weimerskirch H. 2014. Consistency pays: sex differences and fitness consequences of behavioural specialization in a wide-ranging seabird. Biol Lett. 10(10):20140630. https://doi.org/10.1098/rsbl.2014.0630

Wakefield ED et al. 2011. Habitat preference, accessibility, and competition limit the global distribution of breeding Black-browed Albatrosses. Ecological Monographs. 81(1):141–167. https://doi.org/10.1890/09-0763.1
